# Pediatric Posterior Cruciate Ligament Avulsion Fracture of the Tibial Insertion: Case Report and Review of the Literature

**DOI:** 10.1055/s-0037-1605364

**Published:** 2017-08-11

**Authors:** Yannick Hurni, Vincenzo De Rosa, Jorge Gabriel Gonzalez, Mario Mendoza-Sagaon, Flurim Hamitaga, Giorgia Pellanda

**Affiliations:** 1 Department of Pediatric Surgery, Ospedale Regionale Bellinzona e Valli, Bellinzona, Switzerland

**Keywords:** posterior cruciate ligament, avulsion fracture, pediatric

## Abstract

**Background** Posterior cruciate ligament (PCL) avulsion fracture of the tibial insertion is a very rare injury in children. In addition to performing an attentive clinical examination, radiologic studies are fundamental for its correct diagnosis and treatment. Its management may be either conservative or operative. So far, only a few cases treated conservatively have been reported in the pediatric population, with controversial results.

**Methods** We prospectively collected and reviewed clinical and radiographic data of an 11-year-old boy with avulsion fracture of the tibial insertion of the PCL. In addition, we performed a systematic review of the literature available to date.

**Results** We decided to treat the avulsion fracture in a conservative way. The patient has been followed with accurate clinical and radiological follow-up controls until complete recovery.

**Conclusion** Posterior cruciate avulsion fracture is a very rare finding in children, and no definitive indications for its appropriate management exist. With this report, we demonstrate that these fractures can be treated conservatively in selected cases with good results, avoiding potential surgical-related complications.

**Study Design** This is a case report (level of evidence V).

Posterior cruciate ligament (PCL) avulsion fracture of the tibial insertion is a very rare injury in children. In addition to performing an attentive clinical examination, radiologic studies are fundamental for its correct diagnosis and treatment. Its management may be either conservative or operative. So far, only a few cases treated conservatively have been reported in the pediatric population, with controversial results. 1 2 3 4 The present case is singular for the early diagnosis and the conservative management with accurate clinical and radiological follow-up controls until complete recovery.

## Case Report

**Fig. 1:**
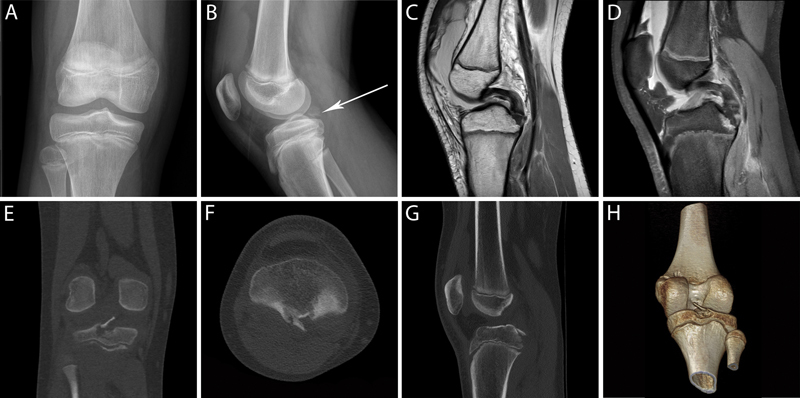
( **A** ) Anteroposterior and ( **B** ) lateral radiograph of the right knee, showing prepatellar intra-articular effusion and isolated avulsion fracture with elevation of the tibial attachment of the posterior cruciate ligament (arrow). ( **C** ) Sagittal T2- and ( **D** ) T1-weighted magnetic resonance imaging sequences showing gross knee effusion, the avulsion fracture and no posterior cruciate ligament injuries. ( **E** ) Coronal, ( **F** ) axial, ( **G** ) sagittal, and ( **H** ) 3D reconstruction computed tomographic images showing fracture of the tibial attachment of the posterior ligament with dislocated tibial fragment measuring 11 × 4 mm and presenting a maximal displacement of 7 mm. 3D, three-dimensional.

An 11-year-old boy was transferred to our institution with right knee pain and swelling after a fall while skiing occurred on the same day. He described the hitting of the frontal aspect of his leg. A physical examination revealed swelling and tenderness on the patella, the lateral aspect of the distal femur, and the medial aspect of the proximal tibia. Because of the pain, the knee could not be examined properly. The range of motion was severely limited by the pain, but a neurovascular examination was normal. Standard radiographs showed prepatellar intra-articular effusion and an isolated avulsion fracture with the elevation of the tibial attachment of the PCL ( Fig. 1A, B ). The diagnosis was subsequently confirmed by computed tomographic scanning, and other bone lesions were excluded ( Fig. 1E–H ). The tibial fragment measured 11 × 4 mm and presented a maximal displacement of 7 mm. No other ligamentous, meniscal, or chondral injuries were observed in a magnetic resonance imaging examination ( Fig. 1C, D ). Because of minimal displacement, we decided to treat the avulsion fracture in a conservative way. The knee was immobilized for 6 weeks, with a long leg cast with 30 degrees of knee flexion. The patient was asked to walk with crutches, avoiding weight bearing. After removing the cast, the patient was allowed to begin gentle range-of-motion activities and weight bearing. The patient was asked to report for regular clinical and radiological controls every 4 to 6 weeks until 3 months after the trauma. No pain or instability was detected during a physical examination, and magnetic resonance imaging showed progressive consolidation of the fracture over time. Subsequently, the patient was allowed to progressively return to sport activities, reporting only rare episodes of knee joint swelling and slight pain during severe exertion. In addition, the patient was asked to fill in the functional knee score of Tegner and Lysholm (1985). 5 With a result of 90/100, the outcome was evaluated as good.

**Fig. 2:**
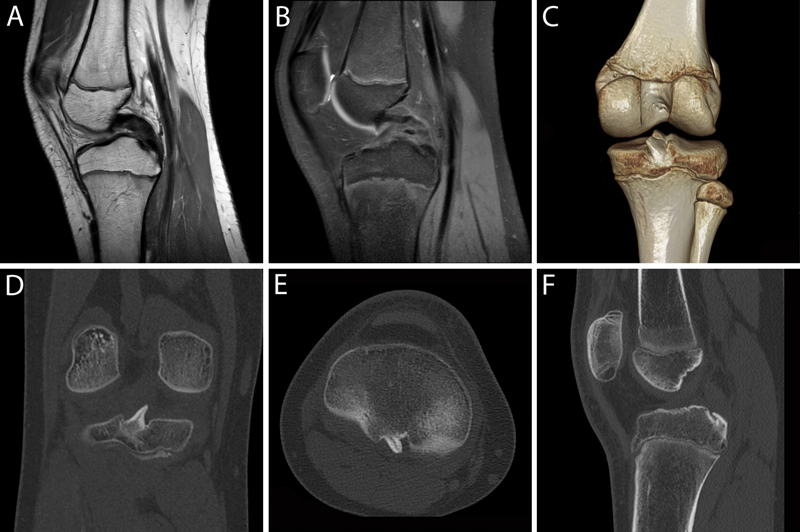
( **A** ) Sagittal T2- and ( **B** ) T1-weighted magnetic resonance imaging sequences of the right knee showing no posterior cruciate ligament injuries. ( **C** ) 3D reconstruction, ( **D** ) coronal, ( **E** ) axial, and ( **F** ) sagittal computed tomographic images showing complete consolidation of the previously fractured tibial attachment of the posterior cruciate ligament. 3D, three-dimensional.

A computed tomographic scanning and a magnetic resonance imaging performed 10 months after the trauma showed complete consolidation of the tibial attachment of the PCL ( Fig. 2A–D ). At this time, the patient has returned to his previous level of physical activity, reporting no complaints. A physical examination revealed no pain or instability of the knee, and muscular strength was comparable to the strength of the contralateral leg.

Fourteen months after the trauma, we re-evaluated the patient for the last time. He was completely asymptomatic, the clinical examination was normal, and the functional knee score of Tegner and Lysholm gave an excellent result with 100/100 points. We decided to discontinue this tight surveillance, and the patient was asked to report for annual controls until complete skeletal growth is achieved.

Written informed consent was obtained from the patient's parents regarding publication of this case report and its accompanying images.

## Discussion

The most important finding of our study was that tibial avulsion fractures of the PCL can be managed conservatively with satisfactory outcomes in pediatric patients. Injuries to the PCL are infrequent at all ages, especially compared with those against the anterior cruciate ligament (ACL). 6 In children, PCL injuries typically involve the avulsion fracture of either the tibial 1 2 7 8 9 10 11 12 13 14 15 16 17 or femoral 3 18 19 20 21 22 23 24 insertion sites, while midsubstance tears are uncommon. 4 25 26 In skeletally immature knees, the ligaments appear to be much stronger than the physis, predisposing to osteochondral avulsions rather than ligamentous tears. 23 27

Sport traumas and traffic accidents are the main reported sources of PCL injuries. PCL injuries can result from a direct anterior blow to the proximal tibia, hyperflexion, or more rarely from sudden hyperextension. 6 28 Forced posteriorly twisting injury has also been observed. 13

PCL injuries generally present with immediate pain, swelling, and limitation of the range of motion. Due to protective reflex muscle spasms and painful mobilization, appropriate knee examination is often difficult. Clinical findings, such as a posterior sag sign, positive posterior drawer, or quadriceps-active test, are classically related to PCL insufficiency. 6 However, they may not be obvious in the acute phase, and only re-examination under anesthesia or several days after the trauma may reveal them. 7 8 12 No clinical sign can differentiate between ligamentous tears and avulsion fractures. PCL avulsion fractures are often associated with other injuries in the same knee. Therefore, an attentive examination of all structures is recommended. 6 Because of potential neurovascular damages, attention must be paid to evaluating the perfusion, sensibility, and motricity of the limb. 19

For these reasons, radiologic studies are fundamental for correct diagnosis and management. Standard radiographs may be useful in the case of a bony avulsed fragment, but one certainly cannot rule out avulsion fractures, especially in the case of incomplete knee ossification. 1 2 11 24 29 Every pediatric patient with suspected PCL insufficiency should benefit from a magnetic resonance imaging screening. Associated intra-articular injuries have often been reported, 4 8 12 15 18 19 20 22 23 and magnetic resonance imaging appears to be the best noninvasive modality in diagnosing them. 30 Specific classifications have been proposed for the more frequently observed avulsion fractures of the ACL, while none has been defined for those of the PCL. 29 In these cases, descriptions of the displacement of the avulsed fragment and of the potential associated injuries are still needed.

In the absence of pertinent guidelines, the management of this injury in children is inspired by indications deriving from ACL avulsion fractures and adult traumatology. If the avulsed fragment is not displaced or is minimally displaced, nonoperative treatment may be suggested. 31 32 Surgical reduction and fixation should be considered in the case of a displaced fragment or conservative treatment failure. 11 32 PCL injuries combined with other ligamentous or meniscal damages should also be treated operatively. 33

The results in the few reported cases of PCL injuries managed conservatively are controversial, with apparent good outcomes in some patients 1 2 and poor ones in others. 3 4 Operative management with open reduction and several types of internal fixations have often been reported with similar good results, 7 8 9 10 11 12 13 18 even in the case of delayed treatment. 17 Arthroscopic fixation has also been reported. 34

While choosing the most suitable management, one should consider the potential risks of both operative and nonoperative treatments. Potential persistent ligamentous insufficiency following conservative treatment may be associated with secondary displacement, pseudoarthritis, and articular degeneration with early osteoarthritis and/or meniscal injuries. 24 35 36 37 On the contrary, iatrogenic physeal injury can be associated with length and angular growth disorders depending on the skeletal maturity and its remaining growth potential. 19 38 39 This surgical risk should be estimated by considering the chronologic, skeletal, and physiologic age of the patient. 25 The desire to return to elite competitions should be questioned in skeletally immature athletes, and operative management should be proposed if it is present. 11 Long-term prognosis after careful treatment of PCL avulsion fractures seems to be good, with typically no or minimal strength, mobility, and stability deficits. 7 10 11 12 18 Young treated athletes report complete returns to sport activities with no restrictions or complaints. 10 11

## Conclusion

PCL avulsion fracture is a very rare finding in children, and no definitive indications for its appropriate management exist. Several authors have recommended the surgical approach, and only a few patients treated conservatively have been reported. With this report, we demonstrate that PCL avulsion fracture can be treated conservatively in selected cases with good results, avoiding potential surgical-related complications. Tight clinical and radiological follow-up is mandatory to achieve the best treatment outcome. Nevertheless, a rapid switch to a surgical approach must be proposed in a case of failure.

## References

[1] Chan A PH Liu K L Ng B KW Occult posterior cruciate ligament avulsion fracture in a paediatric patient: easily missed diagnosis Hong Kong Med J 2012 18 01 73 75 22302919

[2] Frank C Strother R Isolated posterior cruciate ligament injury in a child: literature review and a case report Can J Surg 1989 32 05 373 374 2670163

[3] Sanders W E Wilkins K E Neidre A Acute insufficiency of the posterior cruciate ligament in children. Two case reports J Bone Joint Surg Am 1980 62 01 129 131 7351403

[4] MacDonald P B Black B Old J Dyck M Davidson M Posterior cruciate ligament injury and posterolateral instability in a 6-year-old child. A case report Am J Sports Med 2003 31 01 135 136 12531770 10.1177/03635465030310010701

[5] Tegner Y Lysholm J Rating systems in the evaluation of knee ligament injuries Clin Orthop Relat Res 1985 198 43 49 4028566

[6] Allen C R Kaplan L D Fluhme D J Harner C D Posterior cruciate ligament injuries Curr Opin Rheumatol 2002 14 02 142 149 11845019 10.1097/00002281-200203000-00011

[7] Al-Ahaideb A Posterior cruciate ligament avulsion fracture in children: a case report with long-term follow-up and comprehensive literature review Eur J Orthop Surg Traumatol 2013 23 02 S257 S260 23412271 10.1007/s00590-012-1146-1

[8] Solayar G N Kapoor H PCL tibial avulsion with an associated medial meniscal tear in a child: a case report on diagnosis and management J Pediatr Orthop B 2012 21 04 356 358 21460735 10.1097/BPB.0b013e328346252f

[9] Tsiaviry P Rabemazava A Z Ruzic J C Aliamus A Razafimahandry H J Avulsion fracture of the posterior cruciate ligament in a child: surgical treatment. a case report [in French] Arch Pediatr 2010 17 04 387 390 20219334 10.1016/j.arcped.2010.01.006

[10] Warme W J Mickelson D All-epiphyseal semitendinosus PCL reconstruction in a 10-year-old child J Pediatr Orthop 2010 30 05 465 468 20574264 10.1097/BPO.0b013e3181df863b

[11] Pandya N K Janik L Chan G Wells L Case reports: pediatric PCL insufficiency from tibial insertion osteochondral avulsions Clin Orthop Relat Res 2008 466 11 2878 2883 18648903 10.1007/s11999-008-0373-6 PMC2565031

[12] Ugutmen E Sener N Eren A Beksac B Altintas F Avulsion fracture of the posterior cruciate ligament at the tibial insertion in a child: a case report Knee Surg Sports Traumatol Arthrosc 2006 14 04 340 342 16328460 10.1007/s00167-005-0706-x

[13] Quintart C Elbaum R A case of isolated avulsion fracture of the posterior cruciate ligament in a child [in French] Rev Chir Orthop Repar Appar Mot 1999 85 06 617 620 10575724

[14] Buckley S L Sturm P F Tosi L L Thomas M D Robertson W W Jr Ligamentous instability of the knee in children sustaining fractures of the femur: a prospective study with knee examination under anesthesia J Pediatr Orthop 1996 16 02 206 209 8742286 10.1097/00004694-199603000-00014

[15] Goodrich A Ballard A Posterior cruciate ligament avulsion associated with ipsilateral femur fracture in a 10-year-old child J Trauma 1988 28 09 1393 1396 3418767 10.1097/00005373-198809000-00016

[16] Ross A C Chesterman P J Isolated avulsion of the tibial attachment of the posterior cruciate ligament in childhood J Bone Joint Surg Br 1986 68 05 747 3782236 10.1302/0301-620X.68B5.3782236

[17] Jang K-M Lee S-H Delayed surgical treatment for tibial avulsion fracture of the posterior cruciate ligament in children Knee Surg Sports Traumatol Arthrosc 2016 24 03 754 759 26704790 10.1007/s00167-015-3929-5

[18] Shen H-C Yang J-J Chang J-H Wang S-J Surgical treatment of injury of the posterior cruciate ligament and posterolateral instability in the knee of a 5-year-old child: a case report Am J Sports Med 2007 35 05 831 834 17244904 10.1177/0363546506295081

[19] Hesse E Bastian L Zeichen J Pertschy S Bosch U Krettek C Femoral avulsion fracture of the posterior cruciate ligament in association with a rupture of the popliteal artery in a 9-year-old boy: a case report Knee Surg Sports Traumatol Arthrosc 2006 14 04 335 339 15947912 10.1007/s00167-005-0677-y

[20] Lobenhoffer P Wünsch L Bosch U Krettek C Arthroscopic repair of the posterior cruciate ligament in a 3-year-old child Arthroscopy 1997 13 02 248 253 9127087 10.1016/s0749-8063(97)90164-x

[21] Itokazu M Yamane T Shoen S Incomplete avulsion of the femoral attachment of the posterior cruciate ligament with an osteochondral fragment in a twelve-year-old boy Arch Orthop Trauma Surg 1990 110 01 55 57 2288808 10.1007/BF00431368

[22] Mayer P J Micheli L J Avulsion of the femoral attachment of the posterior cruciate ligament in an eleven-year-old boy. Case report J Bone Joint Surg Am 1979 61 03 431 432 429417

[23] Clanton T O DeLee J C Sanders B Neidre A Knee ligament injuries in children J Bone Joint Surg Am 1979 61 08 1195 1201 511880

[24] Meyers M H Isolated avulsion of the tibial attachment of the posterior cruciate ligament of the knee J Bone Joint Surg Am 1975 57 05 669 672 1150710

[25] Anderson A F Anderson C N Posterior cruciate and posterolateral ligament reconstruction in an adolescent with open physes. A case report J Bone Joint Surg Am 2007 89 07 1598 1604 17606801 10.2106/JBJS.F.00807

[26] Maffulli N Chan K M Bundoc R C Cheng J C Knee arthroscopy in Chinese children and adolescents: an eight-year prospective study Arthroscopy 1997 13 01 18 23 9043600 10.1016/s0749-8063(97)90205-x

[27] Parkkari J Pasanen K Mattila V M Kannus P Rimpelä A The risk for a cruciate ligament injury of the knee in adolescents and young adults: a population-based cohort study of 46 500 people with a 9 year follow-up Br J Sports Med 2008 42 06 422 426 18390920 10.1136/bjsm.2008.046185

[28] Kennedy J C Grainger R W The posterior cruciate ligament J Trauma 1967 7 03 367 377 6024138 10.1097/00005373-196705000-00004

[29] Meyers M H McKeever F M Fracture of the intercondylar eminence of the tibia J Bone Joint Surg Am 1970 52 08 1677 1684 5483091

[30] Sonin A H Fitzgerald S W Hoff F L Friedman H Bresler M E MR imaging of the posterior cruciate ligament: normal, abnormal, and associated injury patterns Radiographics 1995 15 03 551 561 7624562 10.1148/radiographics.15.3.7624562

[31] Torisu T Isolated avulsion fracture of the tibial attachment of the posterior cruciate ligament J Bone Joint Surg Am 1977 59 01 68 72 833178

[32] Coyle C Jagernauth S Ramachandran M Tibial eminence fractures in the paediatric population: a systematic review J Child Orthop 2014 8 02 149 159 24585047 10.1007/s11832-014-0571-6 PMC3965767

[33] Shelbourne K D Davis T J Patel D V The natural history of acute, isolated, nonoperatively treated posterior cruciate ligament injuries. A prospective study Am J Sports Med 1999 27 03 276 283 10352760 10.1177/03635465990270030201

[34] Kwon O S Park M J Kelly J D IV Arthroscopic treatment of a PCL avulsion fracture in a skeletally immature patient Orthopedics 2011 34 02 137 21323274 10.3928/01477447-20101221-34

[35] Torg J S Barton T M Pavlov H Stine R Natural history of the posterior cruciate ligament-deficient knee Clin Orthop Relat Res 1989 246 208 216 2766608

[36] Boynton M D Tietjens B R Long-term followup of the untreated isolated posterior cruciate ligament-deficient knee Am J Sports Med 1996 24 03 306 310 8734880 10.1177/036354659602400310

[37] Wind W M Jr Bergfeld J A Parker R D Evaluation and treatment of posterior cruciate ligament injuries: revisited Am J Sports Med 2004 32 07 1765 1775 15494347 10.1177/0363546504270481

[38] Koman J D Sanders J O Valgus deformity after reconstruction of the anterior cruciate ligament in a skeletally immature patient. A case report J Bone Joint Surg Am 1999 81 05 711 715 10360701 10.2106/00004623-199905000-00014

[39] Robert H E Casin C Valgus and flexion deformity after reconstruction of the anterior cruciate ligament in a skeletally immature patient Knee Surg Sports Traumatol Arthrosc 2010 18 10 1369 1373 19946668 10.1007/s00167-009-0988-5

